# Role of quantitative p16^INK4A^ mRNA assay and digital reading of p16^INK4A^ immunostained sections in diagnosis of cervical intraepithelial neoplasia

**DOI:** 10.1002/ijc.30783

**Published:** 2017-06-01

**Authors:** Nataša Vasiljević, Paul D. Carter, Caroline Reuter, Rhian Warman, Adam R. Brentnall, James R. Carton, Jack Cuzick, Attila T. Lorincz

**Affiliations:** ^1^ Centre for Cancer Prevention, Wolfson Institute of Preventive Medicine, Barts and The London School of Medicine, Queen Mary University of London Charterhouse Square London United Kingdom; ^2^ Centre for Molecular Pathology, Royal Marsden Hospital Sutton United Kingdom; ^3^ Department of Histopathology Charing Cross Hospital London United Kingdom

**Keywords:** p16 RT‐qPCR, p16 Immunohistochemistry, digital H‐score, human papilloma virus, cervical intraepithelial neoplasia

## Abstract

Visual interpretation of cervical biopsies is subjective and variable, generally showing fair to moderate inter‐reader agreement in distinguishing high from low grade cervical intraepithelial neoplasia (CIN). We investigated the performance of two objective p16 quantitative tests in comparison with visual assessment: (*i*) p16‐mRNA assay and (*ii*) digital analysis of sections stained for p16 protein. The primary analysis considered 232 high‐risk human papilloma virus positive (HPV+) samples from diagnostic cervical specimens. A p16 RT‐qPCR (p16‐mRNA assay) was run on mRNA extracted from formalin‐fixed paraffin‐embedded sections. Two p16 immunohistochemistry (IHC) readings, a visual read by a histopathologist (Visual IHC) and a digital read of a high‐resolution scan (Digital IHC), were done on adjacent sections. The worst reviewed CIN grade (agreed by at least two histopathologists) from up to two biopsies and a loop excision was taken, with CIN2/3 as the primary endpoint. Visual IHC attained a specificity of 70% (95%CI 61–77) for 85% (95%CI 77–91%) sensitivity. The four‐point Visual IHC staining area under the curve (AUC) was 0.77 (95%CI 0.71–0.82), compared with 0.71 (95%CI 0.64–0.77) for p16‐mRNA and 0.67 (95%CI 0.60–0.74) for Digital IHC. Spearman rank‐order correlations were: visual to p16‐mRNA 0.41, visual to digital 0.49 and p16‐mRNA to digital: 0.22. The addition of p16‐mRNA assay to visual reading of p16 IHC improved the AUC from 0.77 to 0.84 (*p* = 0.0049). p16‐mRNA testing may be complementary to visual IHC p16 staining for a more accurate diagnosis of CIN, or perhaps a substitute in locations with a lack of skilled pathologists.

## Abstract 2

**What's new?**

Visual examination of cervical tissue obtained by biopsy provides information on morphological changes associated with human papillomavirus (HPV) infection and transformation to precancerous cervical intraepithelial neoplasia 2/3 (CIN2/3). The accuracy of CIN2/3 diagnosis by visual interpretation, however, could be improved through the use of complementary objective tests. This study describes two such tests: quantitative p16 mRNA analysis and digital analysis of immunohistochemical p16‐protein staining. CIN2/3 diagnostic accuracy benefitted especially from the addition of p16‐mRNA testing. Both new approaches could prove useful in locations lacking trained pathologists and could help avoid overtreatment in HPV‐positive women, many of whom recover spontaneously.

**Abbreviations**
AUC: area under curve
CIN: cervical intraepithelial neoplasia
IHC: immunohistochemistry
HPV: human papilloma virus
LLETZ: large loop excision of the transformation zone
p16: p16^INK4A^ protein
RT‐qPCR: real time quantitative polymerase chain reaction

Infection by high risk human papilloma virus (hrHPV) and subsequent failure to attend for cervical screening are the principal factors responsible for high cervical cancer burden. After host exposure to HPV, the virus may become established in tissues as one or more areas of normal‐appearing epithelium which over time remain visually flat or perhaps assume warty features. In early stages of HPV infection, the histopathological examination of biopsies usually appears morphologically normal. They might, however, have various minor changes or sometimes show architecture consistent with cervical intraepithelial neoplasia grade 1 (CIN1). Over a period of years persistent hrHPV infection can transform the affected tissue into high grade intraepithelial disease, either CIN2 or the more worrisome CIN3, which are regarded as precancerous lesions.^1^, ^2^

hrHPV infections and associated low grade lesions are quite common, but almost all spontaneously clear within a few years.^3^, ^4^, ^5^, ^6^ Depending on specific diagnostic criteria, expertise of the pathologist and level of surveillance an estimated 50% of CIN2 may regress without intervention.^7^ The regression rate of CIN3 is incompletely assessed due to ethical considerations, but it is believed that a small percentage of CIN3 ever become invasive.^8^, ^9^ Since the 1960s, in most countries of Europe and North America, cytological screening programs have been implemented with 3–5 yearly screening intervals starting in a woman's mid‐20s. Women with moderate or severe dysplasia on cytology (also called high grade squamous intraepithelial lesion) as well as hrHPV‐positive women with mild or borderline abnormal findings (also called low grade squamous intraepithelial lesion or atypical squamous cells of undetermined significance) are referred to colposcopy.^10^ Some countries are also making the transition to use HPV testing as a primary screen.^11^ The decision on who receives preventative excision treatment is predominantly based on a pathologist's CIN grading of biopsies collected during colposcopy, CIN2 being the most commonly set threshold for intervention.^12^ Although the success of this approach is indisputable,^13^ it is difficult to implement effectively in low‐resource settings. Morphological screening and grading methods are subjective and prone to variability with CIN3 and cancer shown to be the most, and CIN2 the least, reproducible diagnosis.^14^, ^15^ This might lead to unnecessary treatment, which is a concern due to the potential harm to patients and from a monetary perspective. After treatment, women commonly experience substantial pain and discomfort for weeks, however, the main morbidity of treatment is subsequent cervical insufficiency, leading to a higher risk of second and third trimester miscarriage and preterm delivery.^16^ Biomarkers that improve triage of women to colposcopy and provide for a more accurate diagnosis of CIN3 destined to progress are therefore highly desirable.

Elevation of p16^INK4A^ protein, henceforth denoted as p16, in HPV‐positive women may indicate a high‐grade lesion with increased risk of development to cancer.^17^, ^18^ p16 levels are regulated but when hrHPV infection persists a continuous expression of the E6 and E7 oncoproteins inactivates p53 and pRb leading to p16 overexpression. p16 overexpression may therefore be considered a surrogate marker of persistent HPV infection and measurement of its expression allows triage of a higher risk group of hrHPV‐positive women to colposcopy.^19^ In addition, co‐reading of p16 and hematoxylin and eosin (H&E) stained biopsies has been shown to improve observer agreement in the diagnosis of CIN.^20^, ^21^ The reported sensitivity of p16 immunohistochemistry (IHC), however, varies due to difficulties in reproducible reading of the slides, lack of standardized reporting, and uncertainty about the best cut‐off point. Detecting the presence of p16 mRNA by a molecular test rather than staining biopsy sections for the presence of the protein could diminish the subjectivity of p16 testing. In this study, we explore the potential of a novel real time (RT)‐qPCR assay for measuring p16 mRNA expression to improve grading of CIN lesions in a set of formalin‐fixed paraffin‐embedded (FFPE) cervical biopsies from a UK colposcopy referral population.^22^ As a comparison of performance, we also used p16 IHC staining graded by both an expert histopathologist (Visual IHC) and analysed as an H‐Score by an automated digital scanner module (Digital IHC).

## Material and Methods

### Patients

**Figure 1:**
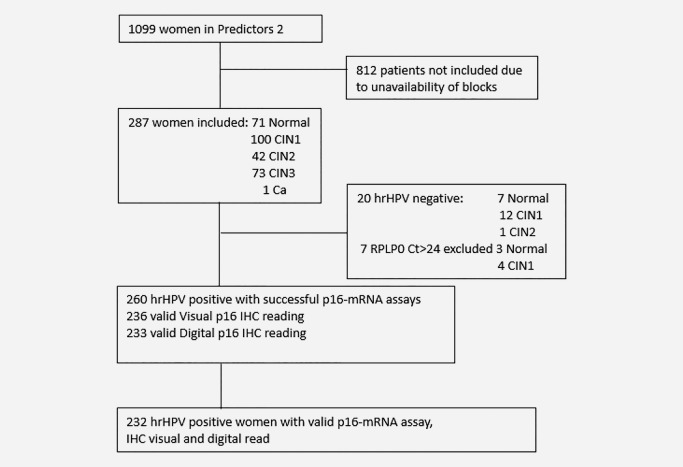
Flowchart showing the number of valid tests results for the women selected for the study.

The Predictors 2 study comprised 1,099 women referred for colposcopy in London between September 2007 and October 2009 following an abnormal cytology result.^22^ Women attended one or two colposcopies. For this study, p16 testing was performed on 287 biopsy specimens that were available from the first colposcopy visit (Fig. 1). Some women were recalled for a second colposcopy and additional biopsies taken where abnormalities were seen. None of the second biopsies were used for p16 testing. Women who met the standard UK criteria for treatment were referred to an expert clinic for large loop excision of the transformation zone (LLETZ). LLETZ tissues as well as second biopsies had histopathological review data available which was considered for the final reference diagnosis in the current study. However, the three p16 tests investigated here were done only on the first biopsies. All women in Predictors 2 received a patient information sheet explaining the study and provided written consent. Approvals were obtained from the relevant local research ethics committees.

### Histopathological diagnosis

In our main analyses, we dichotomized the specimens into a set with the diagnosis of CIN2 or CIN3 (CIN2/3) versus the set with a normal or CIN1 (<CIN2) reference diagnosis. However, additional diagnostic categories were provided by the pathologists and used in some of our secondary analyses. For the Predictors 2 study, H&E stained sections of all study specimens were read by the routine hospital pathologist(s) and reviewed by an expert consultant pathologist. In case of disagreement, diagnosis was arbitrated by a second expert pathologist.^22^ The worst reviewed histology of the second biopsies and LLETZ were retrieved from the Predictors 2 study. For the present study, the first biopsies were also reviewed by a different histopathologist (JRC). To maximise the likelihood of an accurate diagnosis, the worst reviewed histology of the first biopsy was based on JRC's review and the Predictors 2 expert consultant pathologist, while hospital grading was used to arbitrate (Supporting Information methods). The worst concordant diagnosis on any biopsy (first, second and if available the LLETZ surgical specimen) was used to determine the final reference diagnosis for comparisons of the p16 tests.

### HPV testing

HPV status of the 287 patients was determined in the Predictors 2 study by the Hybrid Capture 2 HPV test (Qiagen, Hilden, Germany) and the BD HPV test (Becton Dickinson Diagnostics, Sparks, MD) on exfoliated cervical cells from the concurrent sample.^22^ hrHPV positive samples were defined as specimens positive by either of these two tests.

### p16 IHC for visual and digital reading

Two 5 µm sections were cut from the FFPE first biopsy blocks for H&E and p16 IHC staining. p16 IHC was done using the CINtec p16 Histology assay (Ventana, Tucson, AZ) according to the manufacturer's instructions. The histopathologist (JRC) performed the visual p16 readings, henceforth referred to as Visual IHC, and was blinded to all patient data. Visual IHC was scored using a semi‐quantitative scale with four categories: normal, sporadic, focal or diffuse.^23^ Diffuse staining was the cut‐off point for calling specimens positive.^21^ The p16 stained sections were also scanned using a Panoramic 250 digital slide scanner (3D Histech Kft, Budapest, Hungary). The images were analysed using the Nuclear Quant module (version 1.15.1.9) from which the H‐score (3 × percentage of strongly staining nuclei + 2 × percentage of moderately staining nuclei + percentage of weakly staining nuclei, giving a range of 0–300) was obtained, henceforth referred to as Digital IHC.

### p16‐mRNA assay

Six 10 µm sections from the first biopsies were cut directly into 1.5 ml microcentrifuge tubes and stored at −70°C. The tissue sections were deparaffinised in 320 µl of n‐Hexadecane (Alfa Aesar, Heysham, UK) at 56°C for 3 min. Total RNA was extracted using the RNeasy FFPE Kit (Qiagen) according to the manufacturer's instructions. A Nanodrop 1000 spectrophotometer (Thermo Fisher Scientific, Waltham, MA) was used to quantify RNA and up to 300 ng of DNase‐treated total RNA was reverse transcribed using the QuantiTect Reverse Transcription Kit (Qiagen) according to the manufacturer's instructions. A region of the gene *RPLP0* was selected after optimization experiments as the reference gene. For this test, equal amounts of mRNA from 20 samples were tested on six housekeeping genes obtained from Qiagen: *RPLP0* (Cat. no. QT00075012), *ACTB* (Cat. no. QT00095431), *B2M* (Cat. no. QT00088935), *GAPDH* (Cat. no. QT01192646), *HPRT1* (Cat. no. QT00059066) and *PGK1* (Cat. no. QT00013776). *RPLP0* showed the most stable expression (data not shown). Further, p16 (Cat. no. QT00089964) and *RPLP0* primers were tested for amplification efficiency and specificity using a 10‐fold dilution of Stratagene QPCR Human Reference Total RNA (Agilent Technologies, Santa Clara, CA) and melt curve analysis. Measurement of p16 expression, hereafter called p16‐mRNA assay, was performed on a Rotor‐Gene 6000 (Qiagen) with the following cycling conditions: 95°C for 5 min, then 40 cycles of 5 sec at 95°C and 10 sec at 60°C with a final melting curve measuring step from 60 to 99°C. Two microliters (∼25 ng) cDNA was amplified in a volume of 25 µl containing Rotor‐Gene SYBR Green PCR master mix (Qiagen) and 1× dilution of p16 or RPLP0 primer as per manufacturer's instructions. Each sample was run in duplicate and the average Ct value was calculated. qPCR runs included a four points standard curve, a No‐RT control and a No Template Control in duplicate for both primers. Any sample with a Ct value for *RPLP0* >24 was excluded from the analysis due to insufficient amount of cDNA.

### Statistical analyses

The expression stability of the reference genes was determined using BestKeeper (version 1) software.^24^ The relative expression of p16 was calculated using the ΔΔCt method.^25^ Fold expression of p16 in CIN1, CIN2 and CIN3 was analysed in relation to the median expression in normal biopsies. A Cuzick test for trend and a Mann‐Whitney *U* test were used to assess differences in p16 expression between diagnostic groups. Agreement between the histopathologist reviews was measured with Cohen's Kappa statistic. Correlations were investigated by the Spearman rank correlation coefficient. Logistic regression was applied to determine if p16 methods in combination performed better than Visual IHC alone in the first biopsy, and DeLong confidence intervals for area under the receiver operating characteristic curve (AUC) statistics were obtained. Statistical analyses used GraphPad Prism software (v5.03), Stata v11.2, and R.^26^ A statistical test was accepted as significant if the two‐sided *p* value was <0.05.

## Results

### p16 Expression

**Figure 2:**
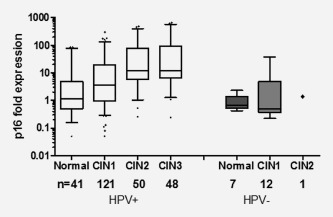
p16‐mRNA expression fold‐difference in the first biopsies of 280 women stratified by worst reviewed histology and HPV status. Fold expression in CIN1, CIN2 and CIN3 is shown in relation to the median expression of 48 normal biopsies. The Cuzick trend test confirmed a significant increasing trend (*p* < 0.0001) with lesion severity in HPV positive women. Higher p16 expression was observed in CIN1 women who were HPV positive compared to HPV negative (*p* = 0.007).

Expression of p16 was successfully measured in 280 of 287 biopsies (Fig. 1) and a trend of increasing p16‐mRNA level was observed with increasing lesion severity *χ* ^2^_1_ = 45.5 (*p* < 0.0001, Fig. 2). There was only one HPV negative patient diagnosed with CIN2+ and the p16‐mRNA level in this woman was low, comparable with women with a normal diagnosis (Fig. 2). A higher level of p16 expression was observed in HPV positive women compared with HPV negative women, with a 7.2‐fold difference observed between the HPV negative and HPV positive CIN1 groups (*p* = 0.007, Fig. 2).

### Agreement between histopathological reviews

The agreement between the pathologists on the first biopsy, where all specimens were read by the two study experts and hospital pathologist(s) for <CIN2 versus CIN2/3, was overall fair to good, with Kappa coefficients of 0.62 (95%CI 0.51–0.73), 0.78 (95%CI 0.69–0.87) and 0.64 (95%CI 0.54–0.75) for the pairwise comparisons (Supporting Information Tables S1–S3). Agreement was, however, poor if all diagnostic categories were taken under consideration, with Kappa coefficients of 0.27 (95%CI 0.17–0.37), 0.37 (95%CI 0.28–0.46) and 0.43 (95%CI 0.34–0.53 (Supporting Information Table S1–S3).

### Comparisons of p16 measures in hrHPV samples

**Figure 3:**
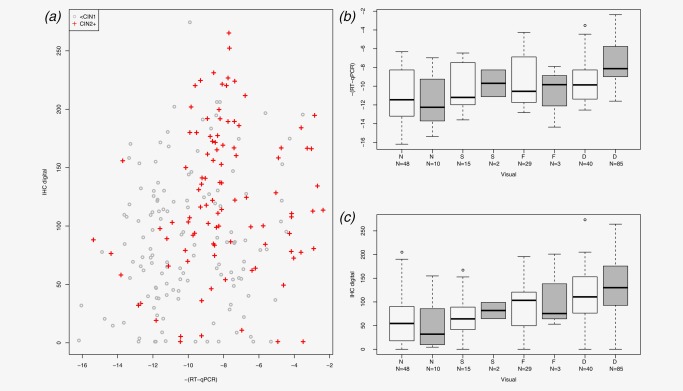
Comparison between three p16 measures. (*a*) p16 mRNA assay (RT‐qPCR) *vs*. Digital IHC (IHC digital), (CIN2+ +; < CIN1 ○); (*b*) p16‐mRNA assay *vs*. Visual IHC staining categories (Visual; N, normal; S, sporadic; F, focal; D, diffuse; gray, CIN2+; white, < CIN2); (*c*) Digital IHC *vs*. Visual IHC staining categories [same format as (*b*)]. [Color figure can be viewed at http://wileyonlinelibrary.com]

Performance of the three p16 assays was analysed in 232 hrHPV positive patients (Fig. 1, Supporting Information Table S4). The association between p16‐mRNA and Digital IHC was weak (Spearman rank correlation 0.22, *p* = 0.0009, Fig. 3 *a*). There was a modest but significant association between the p16‐mRNA assay and Visual IHC (Spearman rank correlation 0.41, *p* < 0.0001, Fig. 3 *b*), as well as between Digital and Visual IHC (Spearman rank correlation 0.49, *p* < 0.0001, Fig. 3 *c*).

Diffuse staining based on Visual IHC had a CIN2+ sensitivity of 85% (95%CI 77–91%) and specificity of 70% (95%CI 61–77%; Table 1).

**Table 1:** The sensitivity and specificity of the visual p16 IHC for CIN2/3 or CIN3 using combinations of the scoring categories, diffuse (D), focal (F) and Sporadic (S) as the cut off

	Cut off	Positive tests (number)	Sensitivity (95% CI)	Positive tests (number)	Specificity (95% CI)
CIN2/3					
	D	85	0.85 (0.77–0.91)	40	0.70 (0.61–0.77)
	D + F	88	0.88 (0.80–0.93)	69	0.48 (0.39–0.56)
	D + F + S	90	0.90 (0.83–0.94)	84	0.36 (0.29–0.45)
CIN3					
	D	58	0.89 (0.79–0.95)	40	0.70 (0.61–0.77)
	D + F	60	0.92 (0.83–0.97)	69	0.48 (0.39–0.56)
	D + F + S	61	0.94 (0.85–0.98)	84	0.36 (0.29–0.45)

**Figure 4:**
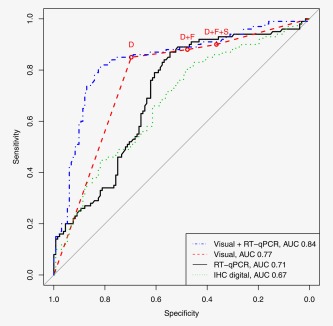
The performance of the different tests to detect CIN2/3 visualized with ROC curves. The circles present the categorical visually read p16 IHC data from Table 1 where the cut offs are based on D (diffuse), D + F (D + focal) and D + F+S (D + F+sporadic) staining. [Color figure can be viewed at http://wileyonlinelibrary.com]

The four‐point Visual IHC staining AUC was 0.77 (95%CI 0.71–0.82), compared with 0.71 (95%CI 0.64–0.77) for the p16‐mRNA assay and 0.67 (95%CI 0.60–0.74) for Digital IHC (Fig. 4, Supporting Information Figure S1). The Visual IHC combined with the p16‐mRNA assay significantly improved the AUC from 0.77 for Visual IHC alone to 0.84 (*p* = 0.0049) for the combination test. In contrast, Digital IHC did not significantly improve the AUC of the Visual IHC test (*p* = 0.21; Table 2 and Fig. 4). A combination of p16‐mRNA and Digital IHC also improved the ability to detect CIN2/3 compared with either test alone (AUC = 0.76, ΔLR‐*χ* ^2^ = 13.8, *p* < 0.0002; c.f. Fig. 3).

**Table 2:** Performance summary of the three tests

	p16 test	LR‐χ2^a^, ^b^	Univariate (*p*‐value)	Univariate AUC (95 CI%)	Combined AUC^c^	ΔLR‐*χ*2 (df = 1)	Addition to Visual IHC (*p*‐value)
CIN2/3	Visual IHC	74.9	<0.0001	0.77 (0.71–0.82)			
	p16‐mRNA	31.8	<0.0001	0.71 (0.64–0.77)	0.84	7.9	0.0049
	Digital IHC	21.5	<0.0001	0.67 (0.60–0.74)	0.81	1.6	0.21
CIN3	Visual IHC	66.8	<0.0001	0.77 (0.71–0.82)			
	p16‐mRNA	26.3	<0.0001	0.71 (0.64–0.79)	0.85	7.4	0.0064
	Digital IHC	28.3	<0.0001	0.73 (0.65–0.80)	0.84	4.7	0.029

a Likelihood ratio *χ* ^2^.

b Univariate LR‐*χ* ^2^ is on 4 df for visual and 1df for RT‐qPCR and IHC digital.

c The combined AUC is for Visual with the quantitative measures fitted using a logistic regression.

## Discussion

This is the first study to measure p16‐mRNA levels and utilise an automated computer‐generated H‐score from digitally scanned images of p16 IHC stained cervical biopsy sections. The performance of these two tests for identifying high grade intraepithelial disease was assessed in comparison with expert visual reading of p16 IHC staining patterns. The aim of the study was to determine the performance of the two quantitative p16 tests univariately and in combination with the Visual IHC results. While p16 staining of biopsies has been shown to improve inter‐reader reliability,^20^, ^21^ this standard method requires a subjective visual interpretation by a trained morphologist. Therefore, objective quantitative methods, such as those investigated here, could complement the expert pathologist H&E reading in routine practice. An objective p16 test would be particularly useful in low to middle‐income regions where there may be a lack of sufficiently trained pathologists. We showed that p16‐mRNA potential to improve the visual diagnosis based on H&E interpretation plus p16 visual scoring and may lead to a more accurate approach to diagnosing CIN2/3 (Fig. 4).

We based our study on a colposcopy referral population to increase power for the endpoint of interest (CIN2/3). Although only 287 of the first biopsies collected in the Predictors 2 cohort were available for p16 testing (Fig. 1), this provided ample power. The histological grade distribution was comparable with the complete colposcopy population. The original biopsies were read by the hospital histopathology team and the sections were subsequently reviewed by two expert histopathologists. Dichotomization of the data at a cut‐off relevant to the decision to treat or not (<CIN2 and CIN2/3), showed good agreement on H&E readings between the histopathologists. However, taking into consideration all diagnostic categories, the agreement was poor (Supporting Information Tables S1–S3). In our study a trend of the expert reviewers downgrading low grade lesion diagnoses was observed, an occurrence that has previously been reported.^27^ Comparing the relative performance of the three p16 approaches tested here, the Visual IHC interpretation with diffuse staining as the cut‐off^21^ yielded the best results (Table 1 and Fig. 4). Other studies based on visual interpretation of p16 staining have shown similar sensitivity and specificity to our study.^28^, ^29^ The novel p16‐mRNA assay performed similarly to the Visual IHC and, at 90% sensitivity, the specificity observed for the mRNA assay was not different than for Visual IHC (Fig. 4). In addition, logistic regression suggested that complementing p16 Visual IHC reading with a p16‐mRNA assay could significantly improve accuracy of diagnosis from an initial AUC of 0.77 to an AUC of 0.84 (*p* = 0.0049) (Table 2 and Fig. 4). The Digital IHC had the weakest performance of all the tests (Fig. 4 and Supporting Information Figure S1). To the best of our knowledge, this utilization of H‐score for p16 expression has not been investigated before. Although such a digital test would avoid the drawback of working with mRNA, a requirement of the p16‐mRNA assay, the scanning equipment is quite expensive and would therefore also be less likely to be applicable in low income regions than use of an inexpensive PCR test.

There was a significant increase in expression of p16 (*p*<0.0001) in CIN2/3 compared to <CIN2 biopsies (Fig. 2), an observation supported by the previous reports that expression of p16 is elevated in higher grade lesions.^17^, ^18^ As there was only one HPV negative CIN2+, no meaningful comparison could be done in this group. The HPV negative CIN1 lesions had significantly lower p16 expression than the HPV positive CIN1 lesions (*p* = 0.007) as would be expected due to lack of HPV interference with the p16 pathway.

Our study has several novel features including the comparisons of the p16‐mRNA assay and the digitally read H‐score to each other and to the visual readings. Although there have been some earlier studies of p16 expression using RT‐qPCR in cervical specimens, all have been on small underpowered sample sets. Upregulation of p16 at both the mRNA and protein level in a small number of carcinomas and cell lines compared to normal tissue was reported in one study,^30^ while another reported a 6‐fold increase in p16 mRNA expression in high‐grade cytology specimens compared to normal cells and HPV‐negative women.^31^

An important limitation of our study was that it was performed on biopsy specimens and there were no comparisons to corresponding exfoliated cervical cells. Unfortunately, a pilot study found that the quality of RNA was too poor in this archived material to obtain valid RT‐qPCR measurements (data not shown). The measurement of p16‐mRNA levels in exfoliated cervical specimens deserves further attention in studies designed to address the mRNA instability problem. It is possible that a combination of p16‐mRNA with other biomarkers such as DNA methylation in exfoliated cell specimens may provide a substantially improved triage test for reducing the number of women requiring referral to colposcopy.

In conclusion, we present two novel approaches, a p16‐mRNA assay and digital reading of p16 IHC, to aid the objective diagnosis of CIN2/3. Application of these measures in a triage test might help to avoid unnecessary treatment in HPV‐positive women. Visual interpretation of p16 IHC is likely to be the best available method, but our data show that use of a p16‐mRNA assay may be complementary and improve the accuracy of the diagnosis. We also envisage the possibility of a future biomarker panel including p16‐mRNA in settings where the diagnostic emphasis is on nucleic acid testing. Analysis of p16 levels alone or in combination with other biomarkers, shows promise to improve diagnosis of cervical intraepithelial neoplasia in locations with a lack of skilled pathologists.

## Conflicts of Interest

None

## Supporting information

Supporting Information
