# Supplementary material for: Role of quantitative p16INK4A mRNA assay and digital reading of p16INK4A immunostained sections in diagnosis of cervical intraepithelial neoplasia
Source: Int J Cancer. 2017 Jun 1;141(4):829–36. doi: 10.1002/ijc.30783 (PMC5949861; doi:10.1002/ijc.30783)

Supplemental Table 1. Agreement between the diagnostic grade provided by the our study expert histopatholgists (JRC) and the consultant expert in Predictors 2 study in the 232 samples. 106 samples had a matching diagnosis. Cohen’s kappa statistics was 0.62 (95%CI 0.51-0.73) for distinguishing <CIN2 from CIN2+, however if all diagnostic categories were taken into account kappa was 0.27 (95%CI 0.17-0.37). UNS samples were not included in Kappa calculations.

|  | UNS | Normal | CIN I | CIN II | CIN III | Total |
| --- | --- | --- | --- | --- | --- | --- |
| UNS | 0 | 1 | 3 | 0 | 2 | 6 |
| Normal | 0 | 24 | 12 | 0 | 0 | 36 |
| CIN I | 1 | 46 | 48 | 14 | 3 | 112 |
| CIN II | 0 | 2 | 10 | 10 | 6 | 28 |
| CIN III | 1 | 4 | 5 | 16 | 24 | 50 |
| Total | 2 | 77 | 78 | 40 | 35 | 232 |

1. UNS = Unsatisfactory specimen or staining, histopathologist could not set the grade.

Supplemental Table 2. Agreement between the diagnostic grade provided by the Predictors 2 study expert histopatholgist (row) and hospital grading (column) in the 232 first biopsies. 118 samples had a matching diagnosis. Cohen’s kappa statistics were 0.78 (95%CI 0.69-0.87) for distinguishing <CIN2 from CIN2+, however if all diagnostic categories were taken into account kappa was 0.37 (95%CI 0.28-0.46). UNS samples were not included in Kappa calculations.

|  | UNS | Normal | CIN I | CIN II | CIN III | Total |
| --- | --- | --- | --- | --- | --- | --- |
| UNS ^1)^ | 0 | 1 | 2 | 2 | 1 | 6 |
| Normal | 0 | 27 | 6 | 1 | 2 | 36 |
| CIN I | 3 | 52 | 49 | 6 | 2 | 112 |
| CIN II | 1 | 2 | 6 | 16 | 3 | 28 |
| CIN III | 1 | 0 | 3 | 20 | 26 | 50 |
| Total | 5 | 82 | 66 | 45 | 34 | 232 |

1. UNS = Unsatisfactory specimen or staining, histopathologist could not set the grade.

Supplemental table 3. Agreement between the diagnostic grade provided by our study expert (JRC) histopatholgist (column) and hospital grading (row) in the 232 biopsies. 133 samples had a matching diagnosis. Cohen’s kappa statistics were 0.64 (95%CI 0.54-0.75) for distinguishing <CIN2 from CIN2+, however if all diagnostic categories were taken into account kappa was 0.43 (95%CI 0.34-0.53). UNS samples were not included in Kappa calculations.

|  | UNS | Normal | CIN I | CIN II | CIN III | Total |
| --- | --- | --- | --- | --- | --- | --- |
| UNS ^1)^ | 1 | 1 | 1 | 2 | 0 | 5 |
| Normal | 1 | 51 | 26 | 3 | 1 | 82 |
| CIN I | 0 | 18 | 37 | 8 | 3 | 66 |
| CIN II | 0 | 5 | 10 | 22 | 8 | 45 |
| CIN III | 0 | 2 | 4 | 5 | 23 | 34 |
| Total | 2 | 77 | 78 | 40 | 35 | 232 |

1. UNS = Unsatisfactory specimen or staining, histopathologist could not set the grade.

Supplemental Table 4: Summary statistics of the mRNA and IHC digital p16 assays.

|  | Status | N | Min | LQ^1^ | Median | Mean | UQ^2^ | Max |
| --- | --- | --- | --- | --- | --- | --- | --- | --- |
| P16-mRNA assay | Normal | 52 | 6.3 | 7.8 | 11.4 | 10.4 | 12.0 | 16.1 |
|  | CIN1 | 80 | 3.5 | 8.2 | 10.2 | 9.8 | 11.8 | 16.2 |
|  | CIN2 | 35 | 2.8 | 6.3 | 8.4 | 8.1 | 9.6 | 15.4 |
|  | CIN3 | 65 | 2.4 | 7.1 | 8.3 | 7.9 | 9.3 | 14.4 |
| Digital IHC | Normal | 52 | 0.0 | 31.5 | 61.1 | 67.8 | 105.9 | 173.6 |
|  | CIN1 | 80 | 0.0 | 53.7 | 87.4 | 96.9 | 137.9 | 273.4 |
|  | CIN2 | 35 | 0.0 | 58.0 | 97.9 | 101.7 | 155.0 | 223.0 |
|  | CIN3 | 65 | 0.0 | 92.8 | 130.1 | 134.5 | 175.8 | 264.3 |

Supplemental Methods

The following describes the process for obtaining the reference histopathological diagnosis in more detail. CIN status was determined from the biopsies (first and second, when available) plus when LLETZ tissues were available. The assignment rule was that the worst reviewed grade from the first and second biopsy (if taken) or the LLETZ (if taken) was assigned as the reference diagnosis. The reviews for lesion grade were as follows.

• First biopsy

• The first biopsy was reviewed by MS and JRC. When both were available (not unsatisfactory) and they agreed, this grade was used (n=106).

• When MS and JRC disagreed (and both reads were available), the hospital grade was used to arbitrate, where possible (n=57 agreed with JRC, n=42 agree with MS)

• For the remaining cases, the grade that was agreed between at least two of MS, JRC and the Hospital review was used for reference assignment (n=18).

• In the remaining women we used the worst grade assessed (n=9).

• Second biopsy.

• The original hospital assessment grade was reviewed by MS and where there was disagreement another expert histopathologistcalled the grade. (retrieved from Predictors 2 study)

• LLETZ

• The grade on the LLETZ tissues was based on the hospital assessment and also reviewed by MS, with another expert histopathologist arbitrating when there was disagreement (retrieved from Predictors 2 study).

Supplemental Figure 1. Histograms showing the distribution of automated measures a) p16-mRNA assay and b) Digital IHC in each diagnostic category.


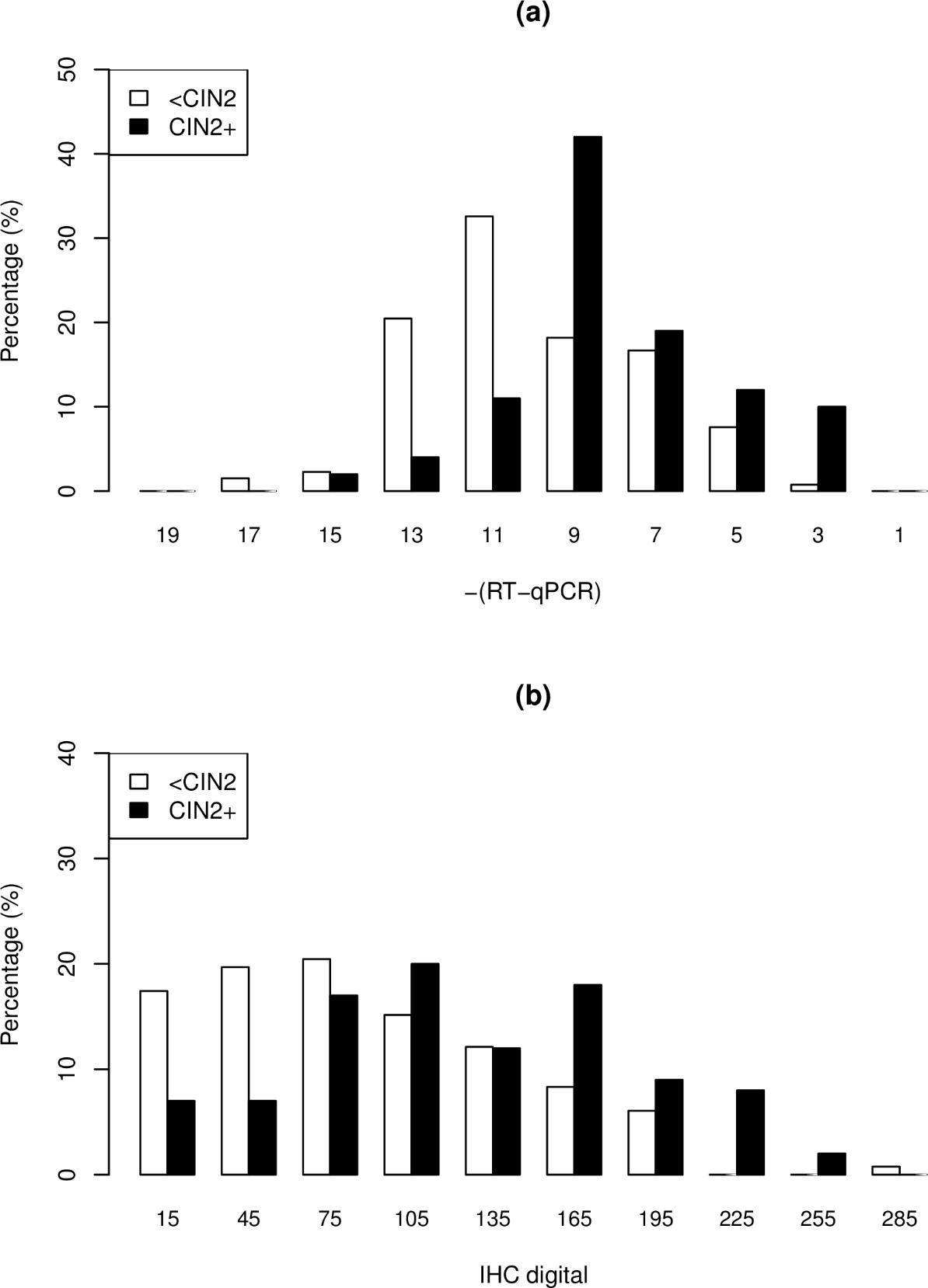

Supplement: Supplementary file 1 — Supporting Information [file IJC-141-829-s001.docx]
